# Developmentally regulated expression, alternative splicing and distinct sub-groupings in members of the *Schistosoma mansoni *venom allergen-like (SmVAL) gene family

**DOI:** 10.1186/1471-2164-9-89

**Published:** 2008-02-23

**Authors:** Iain W Chalmers, Andrew J McArdle, Richard MR Coulson, Marissa A Wagner, Ralf Schmid, Hirohisa Hirai, Karl F Hoffmann

**Affiliations:** 1 Department of Pathology, University of Cambridge, Tennis Court Road, Cambridge, CB2 1QP, UK; 2 Microarray Group, The European Bioinformatics Institute, Wellcome Trust Genome Campus, Hinxton, Cambridge CB10 1SD, UK; 3 Department of Biochemistry, University of Leicester, Lancaster Road, Leicester LE1 9HN, UK; 4 Primate Research Institute, Kyoto University, Inuyama, Aichi 484-8506, Japan; 5 Institute of Biological Sciences, The University of Wales, Aberystwyth, Ceredigion, SY23 3DA, UK

## Abstract

**Background:**

The Sperm-coating protein/Tpx-1/Ag5/PR-1/Sc7 (SCP/TAPS) domain is found across phyla and is a major structural feature of insect allergens, mammalian sperm proteins and parasitic nematode secreted molecules. Proteins containing this domain are implicated in diverse biological activities and may be important for chronic host/parasite interactions.

**Results:**

We report the first description of an SCP/TAPS gene family (*Schistosoma mansoni *venom allergen-like (SmVALs)) in the medically important Platyhelminthes (class Trematoda) and describe individual members' phylogenetic relationships, genomic organization and life cycle expression profiles. Twenty-eight SmVALs with complete SCP/TAPS domains were identified and comparison of their predicted protein features and gene structures indicated the presence of two distinct sub-families (group 1 & group 2). Phylogenetic analysis demonstrated that this group 1/group 2 split is zoologically widespread as it exists across the metazoan sub-kingdom. Chromosomal localisation and PCR analysis, coupled to inspection of the current *S. mansoni *genomic assembly, revealed that many of the SmVAL genes are spatially linked throughout the genome. Quantitative lifecycle expression profiling demonstrated distinct SmVAL expression patterns, including transcripts specifically associated with lifestages involved in definitive host invasion, transcripts restricted to lifestages involved in the invasion of the intermediate host and transcripts ubiquitously expressed. Analysis of SmVAL6 transcript diversity demonstrated statistically significant, developmentally regulated, alternative splicing.

**Conclusion:**

Our results highlight the existence of two distinct SCP/TAPS protein types within the Platyhelminthes and across taxa. The extensive lifecycle expression analysis indicates several SmVAL transcripts are upregulated in infective stages of the parasite, suggesting that these particular protein products may be linked to the establishment of chronic host/parasite interactions.

## Background

Schistosomes are dioecious metazoan parasites of the phylum Platyhelminthes, which are estimated to infect more than 200 million people worldwide, with a further 600 million individuals living in the tropics and sub-tropics at risk of infection. The deposition of schistosome eggs within host tissues and the subsequent immune response elicited are the principal causes of chronic schistosomiasis, which can lead to a range of morbidities such as periportal fibrosis and granulomatous inflammation [1]. Despite the availability of a highly effective chemotherapeutic agent (praziquantel), recent reassessment of disease-related morbidity shows schistosomiasis to be a far greater public health problem than previously estimated [2]. This reappraisal of the impact of schistosomiasis and the potential emergence of praziquantel-resistant strains argues strongly for the identification and characterisation of novel vaccine and drug targets.

*Schistosoma mansoni *is one of three schistosome species that cause the vast majority of human infections and is the most extensively studied in the laboratory. Large-scale sequencing projects have created extensive *S. mansoni *expressed sequence tag (EST) and genomic databases leading to the identification of thousands of new genes, as well as providing a repository of information useful for post-genomic activities [3,4]. In our search for novel chemotherapeutic and immunoprophylactic targets, we have utilised these sequence databases for construction of DNA microarrays to identify gender-associated and developmentally-regulated *S. mansoni *transcripts [5,6]. One interesting finding from these investigations was the identification of two adult male-associated transcripts bearing sequence similarity to the SCP/Tpx-1/Ag 5/Pr-1/Sc7 (SCP/TAPS) family.

Members of the SCP/TAPS family (Pfam accession number no. PF00188; [7]) encode structurally related proteins found throughout the eukaryotic kingdom. All members contain a unique SCP/TAPS protein domain, which varies in length between 120 and 170 amino acids. Tertiary structural studies have demonstrated that this domain adopts a highly conserved α-β-α sandwich conformation [8-13]. The strong conservation of the tertiary structure and of particular residues within the domain have suggested that all SCP/TAPS domain containing proteins share a common biological activity [9]. However, no specific function has yet been ascribed to the SCP/TAPS domain, despite some biological roles having been linked to member proteins within the superfamily. Specifically, superfamily members have been linked to diverse processes including immune responses [14-16], testis/sperm development [7,17], envenomation [18-20] and parasitic nematode invasion of definitive hosts [21-23].

Collectively these data suggest that SCP/TAPS proteins participate in various biological activities across phyla and, as such, warrant further study in the Platyhelminthes as potential modulators of immune function, components of sexual development and candidates for novel vaccine strategies. Towards this end, we present the molecular characterisation of 13 SCP/TAPS family members in *S. mansoni*, hereafter referred to as *Schistosoma mansoni *Venom allergen-like 1–13 (SmVAL1-13). We additionally describe a further 15 members of the SmVAL family predicted in the most recent *S. mansoni *genome assembly [24] and confirm the transcription of eleven of these. Our comparison of the 28 genes provides evidence for the partitioning of the SmVAL family into two groups based on phylogenetic analysis, genomic structure comparison and specific protein feature inclusion. Furthermore, examples of both group 1 and group 2 SCP/TAPS protein types are identified in several metazoan species, supporting an evolutionarily conserved superfamily division across phyla. Analysis of SmVAL1-13 transcription across the schistosome lifecycle demonstrates a range of distinct expression patterns, with a subset exhibiting transcription tightly associated with the invasive stages of the parasite. The proposed functional role of individual SmVAL family members in host/parasite interactions and parasite-specific activities is discussed.

## Results

### cDNA cloning and identification of SmVAL family members

To identify *S. mansoni *SCP/TAPS domain-containing family members, the *Vespula vulgaris *Wasp Venom Allergen 5 (Ves v 5, [Genbank:AAA30333]) protein sequence was used in a tBLASTn search of *S. mansoni *ESTs and Phat [25] predicted genes from version 1 of the publicly available *S. mansoni *genomic database [24]. Thirteen different members with significant sequence similarity to Ves v 5 were identified, twelve originating from EST contigs and one from a Phat predicted gene. After PCR confirmation of the sequences from parasite-derived cDNA, these transcripts were named *Schistosoma mansoni *Venom allergen-like transcripts (SmVAL1-13). The molecular details of these thirteen sequences are shown in Table 1. The available Hidden-Markov Model (HMM) consensus of the SCP/TAPS domain (PF00188; [26]) was subsequently used in a search of the most recent release of the genomic database (version 4). This identified 15 additional family members with complete SCP/TAPS domains, and a further 7 with incomplete SCP/TAPS domains. The molecular details of these sequences are shown in Table 2.

**Table 1:** Molecular Details/Characteristics of SmVAL1-13.

SmVAL^a^	Accession number^b^	*S. mansoni *v4 ID^c^	Protein size (AA)^d^	Gene length (bp)^e^	Signal peptide^f^	Conserved intron phase^g^	Gp1/2^h^
1	AY994061	smp_193680/smp_120240	234	1610	Yes	0/2/0/2	1
2	AY994062	smp_002630	229	1577	Yes	0/2/0/2	1
3	DQ060000	smp_193710	213	1221	Yes	0/2/0/2	1
4	AY994063	smp_002070	181	5861	Yes	0/2/0/2	1
5	DQ269980	smp_179480	270	4399	No**	0/2/0/2	1
6	AY953433	smp_124050.1–3	434	64721	No	1/1	2
7	DQ060001	smp_070240	193	3374	Yes	0/2/0/2	1
8	EU164415	smp_123550	261	3082	Yes	0/2/0/2	1
9	DQ269979	smp_176180	182	6562	Yes	0/2/0/2	1
10	EF421456	smp_002060	170	11934	Yes	0/2/0/2	1
11	DQ151891	smp_012350/smp_128780.2	423	18249	No	1/1	2
12	DQ269978	smp_123540	204	3940	Yes	0/2/0/2	1
13	DQ269977	smp_124060	236	26716	No	1/1	2

(a) Gene names (b) GenBank accession number (c) Schisto GeneDB version 4 systematic ID (d) Protein size (in amino acids) encoded by open reading frame (e) Gene length (in base pairs) derived from Schisto GeneDB assembly 4 (f) Signal peptide prediction with SignalP 3.0 server (*D-score *> 0.43 = yes) double asterisk represents SmVALs with *D-scores *above 0.38 but below 0.43 (g) Conserved Intron Phase derived from Schisto GeneDB assembly 4 (h) Group 1/Group 2 designation

**Table 2:** Molecular Details/Characteristics of SmVAL14-28.

SmVAL^a^	PCR confirmation^b^	*S. mansoni *v4 ID^c^	Protein size (AA)^d^	Gene length (bp)^e^	Signal peptide^f^	Conserved intron phase^g^	Gp1/2^h^
14	Yes	smp_078490	219	1514	Yes	0/2/0/2	1
15	Yes	smp_070250	270	4387	No**	0/2/0/2	1
16	Yes	smp_124070	169	25196	No	1/1	2
17	No	smp_163400	168	13461	No	1/1	2
18	Yes	smp_001890	194	7900	Yes	0/2/0/2	1
19	Yes	smp_123090	186	7381	Yes	0/2/0/2	1
20	Yes	smp_127130	225	9235	Yes	0/2/0/2	1
21	Yes	smp_159290	234	1582	Yes	0/2/0/2	1
22	Yes	smp_139450	219	1505	Yes	0/2/0/2	1
23	Yes	smp_160250	200	1182	Yes	0/2/0/2	1
24	No	smp_141550	195	1189	Yes	0/2/0/2	1
25	No	smp_141560	195	1189	Yes	0/2/0/2	1
26	Yes	smp_154260	182	2727	Yes	0/2/0/2	1
27	Yes	smp_154290	182	2696	Yes	0/2/0/2	1
28	No	smp_176160	182	2713	Yes	0/2/0/2	1

Genes^i ^encoding incomplete SCP/TAPS domains : smp_120670, smp_116210, smp_176170, smp_035980, smp_159280, smp_100560, smp_118160							

(a) Gene names (b) Successful PCR amplification of SmVAL fragment from parasite cDNA (c) Schisto GeneDB version 4 systematic ID (d) Protein size (in amino acids) encoded by open reading frame (e) Gene length (in base pairs) derived from Schisto GeneDB assembly 4 (f) Signal peptide prediction with SignalP 3.0 server (*D-score *> 0.43 = yes) double asterisk represents SmVALs with *D-scores *above 0.38 but below 0.43 (g) Conserved Intron Phase derived from Schisto GeneDB assembly 4 (h) Group 1/Group 2 designation. (i) Schisto GeneDB version 4 systematic ID of predicted genes encoding incomplete SCP/TAPS domains are also listed.

Complete open-reading frames (ORFs) for SmVAL1-13 were predicted by inspection of genomic sequence data, and these representative members (out of the total 28 with full SCP/TAPS domains) were confirmed by PCR amplification from parasite-derived cDNA. Additional full-length mRNA sequences for SmVAL1 (isolated from mixed sex cercariae RNA), SmVAL2 (isolated from mixed sex miracidia RNA), SmVAL4 (isolated from mixed sex cercariae RNA), SmVAL6 (isolated from mixed sex, 7-week adult RNA), SmVAL7 (isolated from mixed sex, 7-week adult RNA) and SmVAL11 (isolated from mixed sex, 7-week adult RNA) were obtained by Rapid Amplification of cDNA Ends (RACE) and these amplicons all contained the confirmed ORFs as well as associated 5' and 3' untranslated regions (UTRs).

Complete ORFs for SmVAL14-28 were not physically obtained, although partial cDNAs for a subset (11 out of 15, Table 2) were identified during reverse-transcription PCR of schistosome cDNA to demonstrate they were indeed expressed. Subsequent bioinformatics analysis presented herein for SmVAL14-28 is based on the predicted full length ORF obtained from *S. mansoni *GeneDB (version 4).

Protein domain prediction using the SMART database [27] identified a single SCP/TAPS domain (SMART accession number: SM00198) in all but one SmVAL transcript, with SmVAL11 containing two. No other SMART predicted protein domains were identified in any of the SmVAL proteins. The SCP/TAPS domains ranged between 135 and 153 AA in length and typically comprised about 60% of the total amino-acid sequence of an individual SmVAL. SmVAL6 is an exception, as it contained a large extension (~295AA) C-terminal to the SCP/TAPS domain.

Prediction of hydrophobic signal peptide sequences using the SignalP program [28] identified twenty-one SmVALs with strong evidence for signal peptides (*D-score *> 0.43), two SmVALs (5 and 15) encoding possible signal peptides (*D-score *> 0.38 but < 0.43) and five SmVALs (6, 11, 13, 16 and 17) showing no evidence of putative signal peptides (*D-score *< 0.15) (Table 1 &2).

### Comparative sequence and phylogenetic analysis of SmVALs

**Figure 1:**
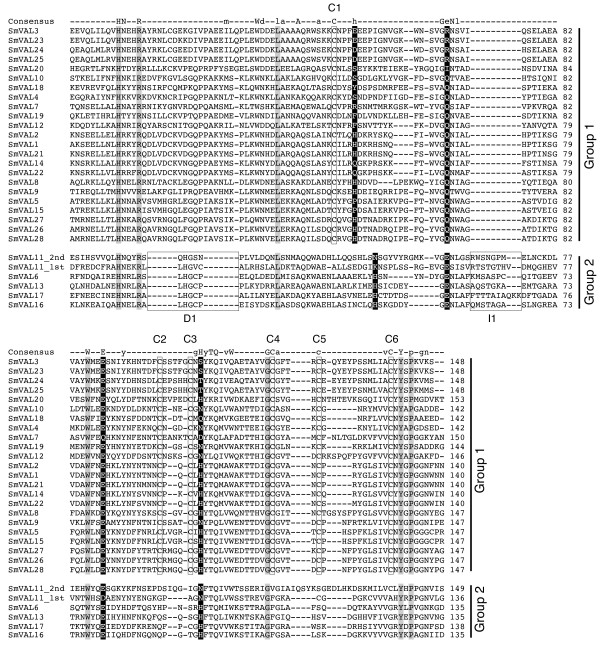
**Alignment of the 29 SCP/TAPS domains present in the deduced amino-acid sequences of SmVAL1-28 demonstrates a clear segregation of the family into two distinct groups**. Group 1 and group 2 members of the SmVAL superfamily are indicated to the right of the MUSCLE-generated alignment. The row labelled 'consensus' represents the SMART consensus sequence of the SCP/TAPS domain (SM00198) where conserved amino acids (50–79%) are indicated by lower-case and highly conserved residues (> 80%) by upper-case letters. The amino acid numbering (indicated at right of alignment) begins at the first residue of the SCP/TAPS domains. Invariant amino acids are highlighted in grey and the group 1 conserved cysteines are boxed and labelled C1 to C6. Amino acids found at the positions of the four residues comprising the putative SCP/TAPS domain active site are coloured in black with white text. The proposed conserved active site contains histidine residues at the first and fourth positions and glutamic acid residues at the second and third positions. The regions of insertion and deletion within the group 2 proteins are boxed and labelled I1 (insertion) and D1 (deletion).

Alignment of the amino acid sequences encoded by SmVAL1-28 revealed significant similarity only over the putative SCP/TAPS domains (Fig. 1). Here, seven residues were found to be invariant among the SmVAL family members (shaded amino acids, Fig. 1). The SMART-derived consensus sequence (indicated on the top of the multiple sequence alignment, Fig. 1) for SCP/TAPS domains indicates five of these seven invariant residues are conserved across phyla (found in 80% or more of superfamily members) and the other two are moderately conserved (found in 50–79% or more of superfamily members). The amino acids (displayed in black boxes) present at the positions of the four residues proposed in structural studies to be the SCP/TAPS domain putative active site (e.g. His72, Glu77, Glu98 and His117 in *Lycopersicon esculentum *P14a [29]) are shown. These putative active site residues show low levels of conservation within the SmVAL family with only SmVAL6, 13, 16 and 17 containing all four residues. Sequence identity over the SmVAL SCP/TAPS domain averages around 34% identity and ranges from very similar (SmVAL1 is over 90% identical to SmVAL2) to weakly related (SmVAL6 has less than 20% sequence identity with SmVAL19).

The multiple sequence alignment also indicated that the SmVAL family could be divided into two groups based on features contained within the SCP/TAPS domain. The first feature is the conservation of one deletion (D1, Fig. 1) and one insertion (I1, Fig. 1) in SmVAL6, SmVAL11, SmVAL13, SmVAL16 and SmVAL17. The second feature is the absolute conservation of six cysteine residues (C1-C6, Fig. 1) in twenty-three SmVAL encoded proteins, which are absent from SmVAL6, SmVAL11, SmVAL13, SmVAL16 and SmVAL17. Together with signal peptide presence/absence, these data support the partitioning of SmVALs into 2 distinct sub-groups (Table 1 &2): group 1, which harbour a signal peptide, contain conserved cysteine residues and lack the D1 and I1 primary amino acid elements and group 2 (SmVAL6, SmVAL11, SmVAL13, SmVAL16 and SmVAL17), which lack a signal peptide, lack the 6 conserved cysteine residues but contain the I1 and D1 regions.

### Phylogenetic analysis of SCP/TAPS proteins

**Figure 2:**
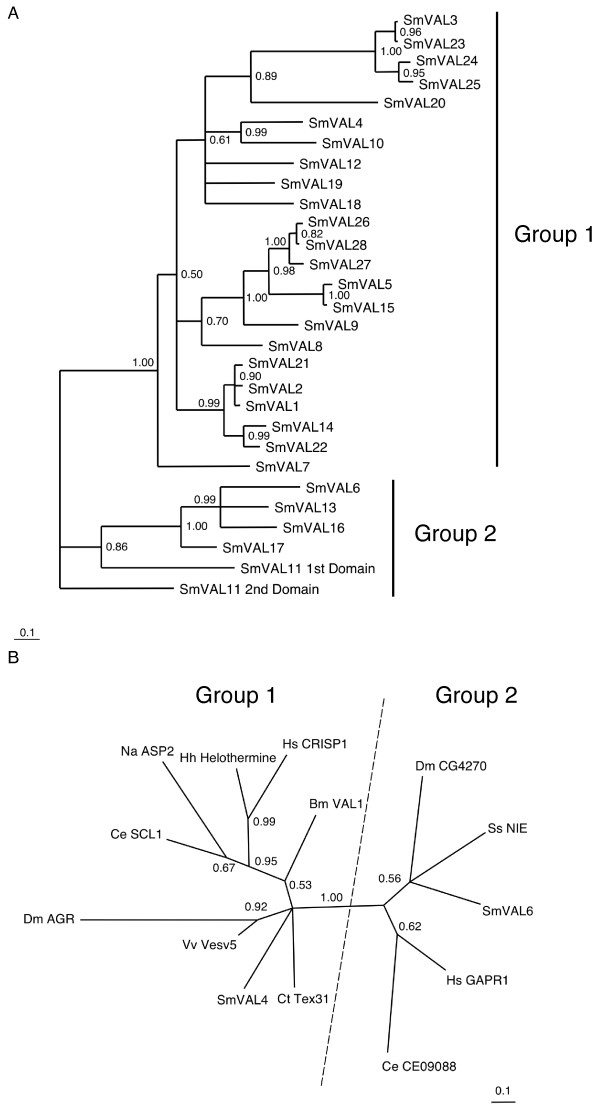
**Phylogenetic analysis of SmVALs and representative metazoan SCP/TAPS proteins reveal a clear and evolutionary distant split between group 1 and group 2 proteins**. Phylogenetic trees were inferred by MrBayes 3.1.2 and illustrated by Treeview as described in *Methods*. Relative branch lengths are indicated, as are the Bayesian posterior probability support values that are greater than 0.5. A) A rooted phylogram is illustrated for the 29 SmVAL SCP/TAPS domain-containing proteins. B) An unrooted phylogram is illustrated for the representative metazoan SCP/TAPS domain-containing proteins. The non-*S. mansoni *protein sequences used have the following GenBank accession numbers: Vv Vesv5 [AAA30333], Dm AGR [AAB92563], Na ASP2 [AAP41952], Ce SCL1 [NP_502502], Ct Tex31 [CAD36507], Bm VAL1 [AAK12274], Ce CE09088 [NP_494312], Dm CG4270 [NP_608663], Hs GAPR1 [Q9H4G4], Ss NIE [AAD46493], Hh Helothermine [2122236A] and Hs CRISP1 [CAC34980]. Branch length represents the number of amino acid changes calculated by the WAG model and is indicated by the scale bars.

To investigate the phylogenetic relationship within the SmVAL family, the predicted 29 complete SCP/TAPS domains were used to construct a phylogram using Bayesian analysis of a conserved (within all 29 SCP/TAPS domains) 84 amino acid region (split across six regions) identified by GBLOCKs [30] (Fig. 2A). The derived phylogram contained two main SmVAL clades (with 100% support) – one harbouring the six SCP/TAPS domains from the group 2 SmVALs and the other containing the 23 SCP/TAPS domains from the group 1 SmVALs (Fig. 2A). These two clades are also well-supported (99% bootstrap value) in the consensus phylogenetic tree constructed by the Neighbor-Joining algorithm (see Additional file 1). Phylogenetic analyses of this type have also been performed for SCP/TAPS domain- containing proteins within *Drosophila melanogaster *[7] and clearly demonstrate that distinct group 1/2 sub-divisions exist within SCP/TAPS family members of other species.

The presence of two distinct groups of SCP/TAPS domains within both *S. mansoni *and *D. melanogaster *led us to question whether this relationship was conserved across diversely related phyla. To study this split further, fourteen representative SCP/TAPS family members from species within Platyhelminthes, Nematoda, Arthropoda, Gastropoda and Chordata were used for extended phylogenetic analysis (Fig. 2B). The derived phylogram again contains two main clades (100% support), with the representative SmVAL group 1 member (SmVAL4) and the representative *D. melanogaster *group 1 member (DmAGR) found in one clade whereas the group 2 representatives of these species are clustered in the other clade (SmVAL6 and Dm CG4270). An additional larger analysis examining SCP/TAPS family members from 44 (out of 56 queried) eukaryotic genomes using different phylogenetic methods demonstrated the wide-scale presence and independent segregation of group 1 and group 2 proteins throughout the metazoan subkingdom (data not shown) and further supported this observation.

### Homology modelling of SmVALs

**Figure 3:**
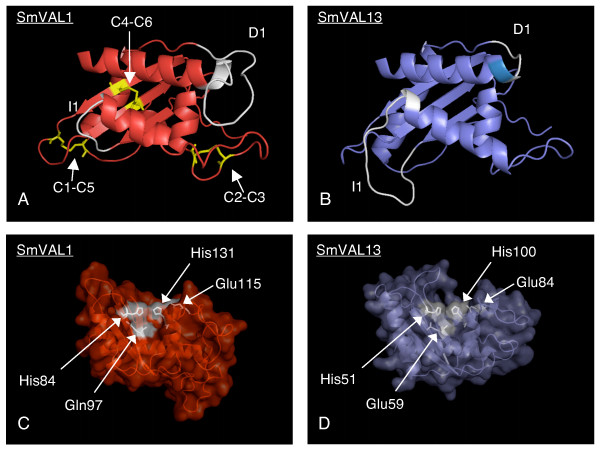
**Homology modelling of representative group 1 and group 2 SmVAL proteins demonstrates the SCP/TAPS domain of both groups possess an α-β-α tertiary structure with a large surface cleft containing the proposed family active site**. Models were constructed for all SmVAL SCP/TAPS domains using MODELLER as described in *Methods *and are represented here by SmVAL1 (group 1, panels A and C) and SmVAL13 (group 2, panels B and D). A cartoon representation of the core α-β-α sandwich structure is coloured in red for SmVAL1 (A) and blue for SmVAL13 (B). Conserved cysteines and the disulphide bonds are coloured yellow, with the cysteines involved in the formation of each disulphide bond labelled C1-C6. The two regions I1 (insertion) and D1 (deletion) are coloured white on each structure. Panel C (SmVAL1) and D (SmVAL13) are semi-transparent visualisations of the protein surface with the four putative active site residues shown in white.

To explore the tertiary structural characteristics within the SCP/TAPS domain of the SmVAL family, we created homology models of the sequence verified SmVAL1-13 members using MODELLER [31] (Fig. 3). Two templates, *Necator americanus *Na-ASP 2 [PDB:1u53] and *L. esculentum *p14a [PDB:1cfe] were used to model the group 1 SmVAL SCP/TAPS domains (represented in Fig. 3A by SmVAL1), and one template, *Homo sapiens *GAPR-1 [PDB:1smb] was used to model the group 2 SmVAL SCP/TAPS domain (represented in Fig. 3B by SmVAL13). Importantly, these derived models suggested that all SmVAL SCP/TAPS domains will adopt the α-β-α sandwich conformation common to all superfamily members across phyla. Secondly, these models demonstrated that the six conserved cysteine residues within the group 1 SmVALs are capable of forming three disulphide bonds (yellow residues, C1-C5, C2-C3, C4-C6, Fig. 3A). Thirdly, these models illustrated that the SmVAL group 2-specific insertion and deletion domains are both found in the loop regions between the α-β-α secondary structural elements (white regions, I1 and D1, Fig. 3). Finally, homology model surface analysis demonstrated that both SmVAL1 and SmVAL13 SCP/TAPS domains possess the large cleft proposed to contain the active site [29] (Fig. 3C and 3D). Within the cleft, the SmVAL1 model (Fig. 3C) contains three of the four putative active site residues (His84, Glu115, His131), with the first glutamic acid of the active site being substituted by a glutamine (Gln97). In the SmVAL13 model, all four proposed active site residues (His51, Glu59, Glu84 and His100) are present on the surface within the cleft (Fig. 3D). In both models, these four residues are found occupying a similar orientation to orthologous residues identified in previous crystallization studies of SCP/TAPS family members [12,13].

### SmVAL gene characteristics

SmVAL1-28 genomic sequences were obtained from the *S. mansoni *genome database [24] by comparing the laboratory derived cDNA sequences to the assembled genomic sequences (version 4). To verify the current assembly, complete genomic sequences for SmVAL1, SmVAL2, SmVAL3, SmVAL5 and SmVAL7 and partial genomic sequences for SmVAL4, SmVAL6 and SmVAL8-13 were obtained by PCR amplification of *S. mansoni *genomic DNA. When compared, these laboratory-derived sequences confirmed the assembled genomic sequences for each SmVAL gene from *S. mansoni *GeneDB. The gene sizes of SmVAL1-28 ranged from 1182 bp (SmVAL23) to ~60 kbp (SmVAL6) (Table 1 &2). The gene structures of the SmVAL family members were investigated by aligning the cDNA sequences with the gene sequences from the genomic database. With three exceptions, all exon/intron junctions conformed to the consensus (GT/AG) splice donor/acceptor sequences for eukaryotes. Exon 10 of SmVAL6 and exon 3 of SmVAL11 use a 5' GC splice donor, and in exon 38 of SmVAL6 the splice donor has been replaced by AC. Given that cDNA sequences of SmVAL6 and SmVAL11 are found to be spliced at these locations, these divergent splice sites appear to be functional.

**Figure 4:**
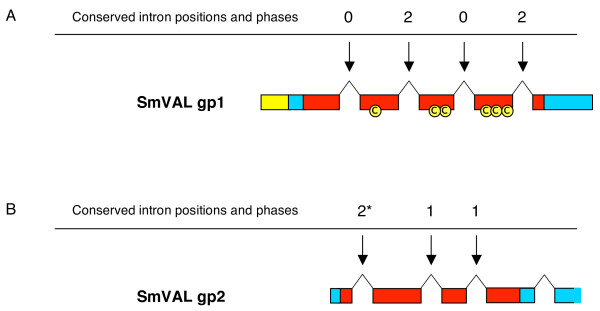
**SmVAL group 1 and group 2 gene structures are unique and likely evolved from a common ancestral gene within the genus**. All SmVAL gene structures were manually assembled by comparison of laboratory derived cDNA sequences to assembled genomic sequences (SchistoGeneDB, version 4). A) Representative group 1 SmVAL gene structure and B) representative group 2 SmVAL gene structure over the SCP/TAPS encoding exons. Exons are represented by boxes with introns represented by lines. The phases of introns are indicated above the arrows. Exons are coloured to show regions encoding important protein features; predicted signal peptides (yellow), SCP/TAPS domain (red) and the remaining ORF (blue). The position of the six conserved cysteines in the group 1 SmVAL proteins is indicated. The single asterisk (*) indicates an intron conserved in all group 2 genes except SmVAL11.

As with the group 1/group 2 split in conserved cysteines, signal-peptide predictions and phylogenetic tree, the group 1 SmVALs differed from the group 2 SmVALs in gene structure (Fig. 4). All group 1 SmVAL genes possess five exons interrupted by four introns (Fig. 4A). The positioning and phase of each of the four introns in relation to the coding sequence of the genes was common to all (Fig. 4A). The group 2 SmVAL genes varied in the number of exons, but within the coding region for the SCP/TAPS domain, two intron positions and their phases were conserved throughout all members (Fig. 4B).

**Figure 5:**
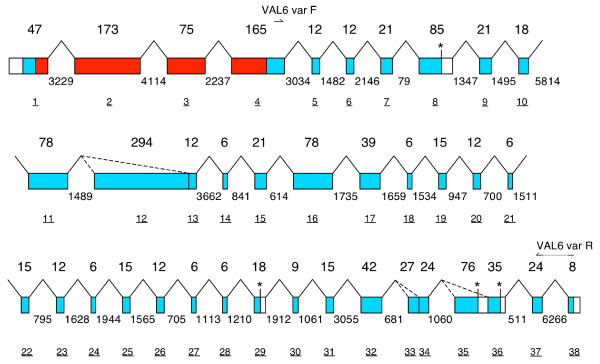
**SmVAL6 genomic structure analysis reveals a highly complex gene composed of 38 exons**. SmVAL6 exons are represented by boxes, with exon length (in base pairs) given above each box. Introns are represented by lines, with intron length (in base pairs) shown below each line. Exons are coloured to indicate: SCP/TAPS domain (red), untranslated region (white) and remaining ORF (blue). Each successive exon has been ascribed a number (underlined numbers below the exons). Exons that introduce a premature stop codon are highlighted with an asterisk and dashed lines represent separate exons created by competing 3' splice sites. The exon specific annealing positions of primers VAL6 var F and VAL6 var R, used for developmentally-regulated alternative splicing analysis, are indicated.

Despite the conserved intron/exon structure (within the group 2 SmVALs) comprising the SCP/TAPS domain of SmVAL6, the intron/exon structure coding for the C-terminus of SmVAL6 is notable in its complexity. Unlike all other family members, the large and extended genomic DNA (gDNA) region 3' of the SCP/TAPS domain is composed of numerous small exons. In total, the gene encoding the SmVAL6 1302 bp transcript is comprised of 38 exons, with 17 smaller than 20 bp (Fig. 5).

### SmVAL genomic clusters

**Figure 6:**
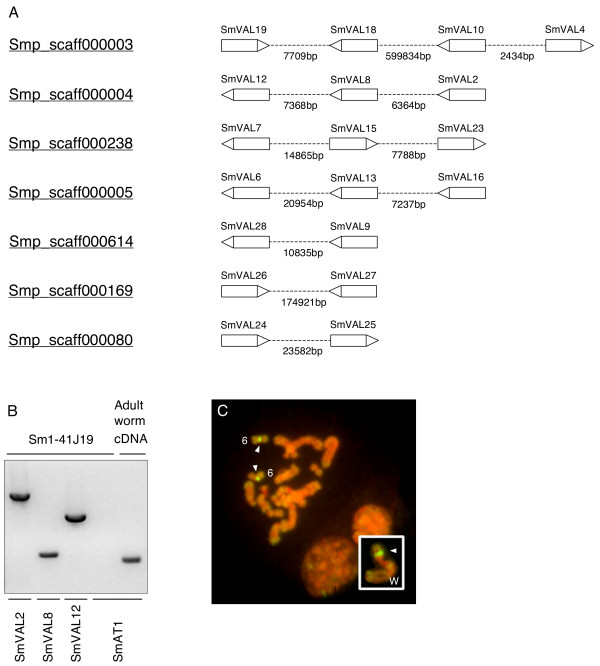
**SmVAL gene clusters exist throughout the genome with SmVAL2, SmVAL8 and SmVAL12 genetically linked to chromosome 6 and W**. A) Genomic regions containing two or more SmVAL genes were identified from interrogation of the current Schisto GeneDB v4 assembly. Schisto GeneDB v4 scaffold ID for each region is shown. SmVAL genes are shown as labelled boxes with the direction of transcription indicated by a triangle at the stop codon. The genomic sequence between SmVAL genes is represented with a dashed line and the length in base pairs shown below. B) Genomic linkage of SmVAL2, 8 and 12 was established by PCR amplification of gene specific regions from BAC clone Sm1-41J19. SmAT1 (alpha tubulin, M80214) was not contained on the BAC clone and was only amplified from adult worm cDNA. C) FISH analysis indicates positive signal (arrowheads) for Sm1-41J19 on chromosome 6 and W (W in inset). Bar indicates 10 μm.

Version 4 of the *S. mansoni *genomic assembly predicted several SmVAL gene clusters to be spatially linked to distinct chromosomal regions (Fig. 6A). To experimentally verify the existence of these distinct SmVAL gene clusters, BAC clones spanning the genome assembly were sought for independent analysis. One BAC clone, Sm1-41J19, was subsequently identified and PCR analysis of its DNA sequence confirmed that the SmVAL2/SmVAL8/SmVAL12 gene cluster was indeed present (Fig. 6B). Subsequent fluorescence in situ hybridisation (FISH) localized Sm1-41J19 (containing SmVAL2/SmVAL8/SmVAL12) to the long arm of chromosome 6 and the short arm of chromosome W (Fig. 6C).

### Developmental expression of SmVALs

**Figure 7:**
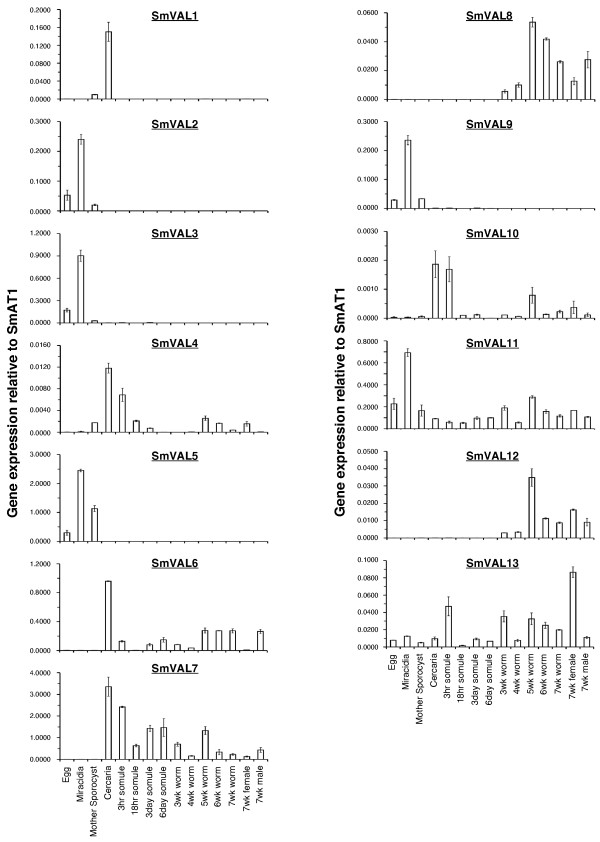
**SmVAL transcription throughout the schistosome life cycle includes both developmental and constitutive patterns**. Total RNA from indicated life-stages was obtained as described in *Methods *and utilized for real time quantitative PCR analysis to determine SmVAL1-13 transcript abundance. For each SmVAL transcript, a bar graph is displayed indicating relative abundance (compared to SmAT1) across the *S. mansoni *lifecycle. On the *x*-axis, each specific life-stage cDNA being tested is indicated. The *y*-axis represents the ratio of SmVAL gene expression relative to that of SmAT1 (reference gene). Data are presented as mean ratios (+/- standard deviation) from technical duplicates.

Lifecycle expression profiles of the sequence-verified SmVAL family members (SmVAL1-13) were obtained by real-time RT-PCR using cDNA created from the mRNA of selected *S. mansoni *life-stages (Fig. 7). Here, distinct examples of normalised SmVAL gene expression throughout the lifecycle were revealed including those displaying either developmental or ubiquitous patterns. One developmentally regulated pattern of SmVAL transcription associated with invasion of the definitive host was found for genes encoding SmVAL1, SmVAL4 and SmVAL10. Specifically, SmVAL1 expression was minimal in all stages examined except mother sporocyst and cercaria, with expression levels estimated to be 15-fold higher in cercaria compared to mother sporocyst. SmVAL4 and SmVAL10 also displayed peak expression in stages associated with vertebrate invasion (cercaria and 3 hour schistosomule) although other life stages show minimal expression.

The real time PCR results also revealed a second pattern of developmental regulation with SmVAL2, SmVAL3, SmVAL5 and SmVAL9 showing expression associated with the invasion of the intermediate host. Specifically, SmVAL2, SmVAL3, SmVAL5 and SmVAL9 transcription was restricted to egg, miracidia and sporocyst life stages, with the miracidial stage showing an expression peak for all four gene products. A third type of developmentally regulated transcriptional profile was observed for SmVAL8 and SmVAL12 mRNAs. Here, both genes were up-regulated during worm maturation with peak levels of transcription occurring in the 5-week worm. Finally, transcription of SmVAL6, SmVAL7, SmVAL11 and SmVAL13 appeared to be far less restricted, with measurable levels of mRNA found in most life stages examined. SmVAL6 and SmVAL7 were minimally transcribed in the egg, miracidial and mother sporocyst stages, dramatically up-regulated in the cercaria and intermediately expressed in all subsequent somule/worm samples. Ubiquitous (but variable) transcription for SmVAL11 and SmVAL13 are seen in all parasite samples with expression peaks observed in the miracidial (SmVAL11) and adult female worm (SmVAL13) life stages.

### Developmentally regulated alternative splicing of SmVAL6

**Figure 8:**
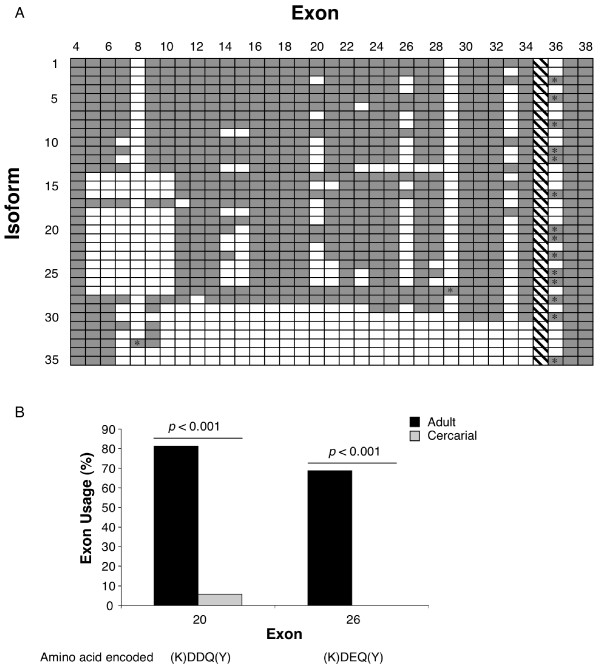
**SmVAL6 exhibits developmentally-regulated alternative splicing **. Sixty-seven individual SmVAL6 cDNA clones (spanning exons 4–38) were isolated and sequenced from parasite material: 32 clones were derived from 7-week mixed-sex adult worm cDNA and 35 clones from mixed-sex cercarial cDNA (see *Methods*). A) Thirty-five distinct SmVAL6 isoforms were identified from the sixty-seven clones sequenced. Columns represent exon number (as described in Fig. 5) and rows indicate the 35 detected isoforms. Filled, grey boxes represent presence of exon, whereas empty, white boxes represent absence of exon in each of the 35 isoforms. The presence of an asterisk indicates an exon encoding a premature stop codon. The diagonal lines in the exon 35 column indicate that this exon has been detected in previous studies but was not observed in any of the transcripts in this experiment. B) Presence of exons 20 and 26 is associated with adult cDNA clones. Frequency of exon 20 and 26 usage in adult (black bars) and cercariae clones (grey bars) is shown with the χ^2 ^test *p *values indicated. The amino acids encoded by each exon are shown below, with amino acids spanning splice acceptor/donator sites indicated in parentheses.

In the process of cloning SmVAL6, and likely due to its genomic complexity (Fig. 5), many different individual full-length ORFs were identified. Interestingly, all sequence variability was limited to the region C-terminal to the SCP/TAPS domain. We used primers (indicated in Fig. 5) designed to amplify this specific region, both to further examine the level of SmVAL6 variation and to compare the diversity of SmVAL6 products expressed in different developmental stages. Analysis of restriction digests from SmVAL6 PCR amplicons throughout the life cycle showed that the greatest qualitative differences in RFLP (restriction fragment length polymorphism) patterns existed between cercarial and 7-week adult worms (data not shown). Therefore, clones derived from cercarial and adult 7-week mixed-sex cDNA were subsequently isolated and sequenced. Analysis of the randomly-selected clones, 32 from 7-week adult worm samples and 35 from cercarial samples, showed that the SmVAL6 transcript is highly variable in both developmental stages (Fig. 8). From the 67 clones sequenced, 35 separate isoforms were observed (Fig. 8A). The variation observed was found to be due either to the absence/presence of exons or alternative 3' splicing within an exon with none of the single nucleotide polymorphisms detected shared by more than one clone. No isoform was predominant; the most abundant isoform accounting for only 7 clones (data not shown). To represent the different isoforms identified in this study, each exon found in the SmVAL6 gene was numbered (according to Fig. 5) and each isoform was scored for the presence or absence of each exon (Fig. 8A). Using the χ^2 ^test, statistically significant developmental regulation of exon expression was found for two exons – 20 (p < 0.001) and 26 (p < 0.001) (Fig. 8B). Interestingly, these two exons were found specifically associated with SmVAL6 transcripts expressed by the adult 7-week life stage and encode very similar amino acid pentamers (KDDQY & KDEQY, respectively) (Fig 8B).

## Discussion

Critical to the development of novel schistosome intervention strategies is the identification of exploitable parasite gene products that functionally participate in important biological niches such as host invasion, chronic host/parasite interactions or immunomodulation. With the availability of genomic information to support the use of transcriptomic and proteomic tools [32], the discovery of exploitable schistosome gene products can now proceed at a previously unattainable rate. We have capitalised on this wealth of information to lead a study culminating in the molecular description of twenty-eight *S. mansoni *SCP/TAPS family members, designated SmVAL1-28. While the exact function of each SmVAL family member is currently unknown, the information provided in our study suggests potential roles in larval penetration, host immune response modulation and adult worm development.

One of the most striking observations in this study, mirroring a recent *Drosophila melanogaster *investigation [7], was the clear segregation of the SmVAL superfamily into two distinct phylogenetic groups (group1/group 2, Fig. 2A). Importantly, we now show this segregation was not solely limited to the Arthropoda and Platyhelminthes, as additional phylogenetic analyses demonstrated that the group 1/group 2 partition to be particularly well conserved and present in other phyla including Nematoda and Chordata (Fig. 2B). SmVAL amino acid features (e.g. signal peptides, cysteine residues, I1 and D1 regions; Table 1, Table 2, Fig. 1 and Fig. 3) also segregated well into the group 1/group 2 partition and further strengthened the phylogenetic predictions. Evidence to support that these group-specific amino acid features may impart distinct SmVAL tertiary structural characteristics originates from the solved crystal structures of both group 1 [8,9,11-13,33] and group 2 [10] members. Although similar α-β-α core SCP/TAPS domains can be formed, the domains found in the group 1 proteins are stabilised by disulphide bonds (mediated by the conserved cysteines), whereas the domains formed in group 2 proteins do not need these structural features. Disulfide bonds are frequently found in extracellular proteins, but only rarely in intracellular proteins. Therefore, the structural differences found in group 1 and group 2 proteins are in accordance with different localisations/fates for the group 1/group 2 SCP/TAPS domain containing proteins. This contention is supported by localization studies involving several SCP/TAPS domain-containing proteins. Golgi-associated PR-1 protein, GAPR-1 (which, like the group 2 SmVALs, lacks a signal peptide and conserved cysteines) demonstrated intracellular association with golgi membranes [34,35] whereas Tex31, Allurin and Ac-ASP 2 (which, like the group 1 SmVALs, contain both signal peptides and conserved cysteines) are detected in extracellular locations [18,36,37]. Therefore, irrespective of the function(s), one potential advantage of encoding two distinct SCP/TAPS domains within the same organism is the ability to have one protein type specialised for the extracellular environment (with signal peptide for secretion and stabilising disulphide bonds) and another type specialised for functioning intracellularly. Indeed, diversification of this kind has recently been reported for the annelid globin gene family [38].

Comparison of the group 1 (SmVAL1) and group 2 (SmVAL13) SCP/TAPS domain homology models showed similar overall structures except for subtle differences in two of the loop regions (I1 and D1). Both proteins also contain the large cleft (Fig. 3C and 3D) which is thought to be the location of the SCP/TAPS domain active site [29]. Four of the amino acids (two histidines and two glutamic acids, positions indicated in Fig. 1 as black boxes) present within the cleft have been proposed to form an active site of undefined activity due to their location, surface accessibility and evolutionary conservation [29]. Interestingly, all group 2 SmVAL proteins except SmVAL11 contain all four residues (Fig. 1) within this surface cleft (e.g. SmVAL13, Fig. 3D) while none of the group 1 SmVAL proteins contain all four residues (Fig. 1) within this cleft (e.g. SmVAL1, Fig. 3C). This suggests that any potential activity (mediated by these four residues) across the family could be variable and therefore, diverse functions within the SmVALs would be substantial.

SmVAL group-specific differences are also observed in the expression of gene products during the parasite's life cycle (Fig. 7). The real-time PCR analysis of SmVAL1-13 showed that many group 1 genes displayed developmentally-regulated expression patterns while all examined group 2 genes demonstrated far less restricted transcription profiles. Group 1 members SmVAL2, SmVAL3, SmVAL5 and SmVAL9 all share a similar expression pattern with transcription peaking in miracidia, suggesting roles for these gene products in the free-living stage or in intermediate host invasion. The minimal presence of these transcripts in the egg stage may be due to our use of both immature & mature (miracidia-containing) egg material. However, recent proteomic studies of *S. mansoni *egg secretions have detected all four of these SmVALs, suggesting a potential secondary role in the egg [39]. The role of SmVAL2, SmVAL3, SmVAL5 and SmVAL9 secretion from unhatched eggs is currently being addressed as part of ongoing experiments into the protein secretion and localisation of SmVALs.

Unlike SmVAL2, SmVAL3, SmVAL5 and SmVAL9, the developmental expression profiles of group 1 members SmVAL1, SmVAL4, and SmVAL10 are linked to definitive host invasion – SmVAL1 to the cercarial stage; SmVAL4 and SmVAL10 to both cercariae and 3-hour schistosomula (Fig. 7). The SmVAL4 and SmVAL10 transcriptional results are corroborated by proteomic studies showing that these gene products (as well as SmVAL18) are indeed present in *S. mansoni *schistosomule secretions [40] further supporting a role in definitive host invasion. Overall, the presence of SmVAL2, SmVAL3, SmVAL4, SmVAL5, SmVAL9, SmVAL10 and SmVAL18 proteins in secreted samples supports the signal peptide predictions for these proteins and, on a more general level, suggests that many or all of the SmVAL group 1 proteins are released from the schistosome and capable of interacting with their immediate environment.

Release of group 1 SCP/TAPS proteins during parasitism may not be limited to the trematodes as *Ancylostoma caninum *(Ac)-ASP 1-6, *Haemonchus contortus *(Hc)-24 & 40, *Ancylostoma ceylanicum *(Ay)-NIF and Ac-HPI are also secreted during nematode infective processes [41-44]. Given the biological activities of the hookworm proteins Ay-NIF (neutrophil inhibitory factor) and Ac-HPI (platelet inhibitory protein), it is tempting to speculate that some secreted group 1 SmVALs may have similar immunomodulatory roles acting, like NIF and HPI, via interactions with integrins. Other potential functions for group 1 SmVALs that may confer selective advantages during parasite invasion may relate to protease activity (Tex31 and RTVP-1) or protease inhibition (P25TI) [18,45,46]. Studies are ongoing to ascertain SmVAL group 1 specific functions.

While all secreted group 1 SmVALs may indeed participate in host/parasite interactions, there is also evidence suggesting that SmVAL1, SmVAL4, SmVAL10 and SmVAL18 (released into the definitive host) may be targets of the adaptive immune response. For example, the hymenopteran venom component Ves v5 is a specific SCP/TAPS allergen capable of activating basophils via an IgE dependent mechanism in sensitised patients [47,48]. Furthermore, human IgE reactivity against the hookworm ASP-2 protein (component of the vertebrate infective L3 stage excretory/secretory products [21,36]) is positively associated with light hookworm infections in heavily exposed endemic populations [49]. This data, as well as successful vaccine experiments in animal models [49-52], has led to ASP-2 becoming a major human hookworm vaccine candidate [53]. Together, these observations suggest that secreted group 1 SmVALs (and possibly group 2 SmVALs [54]) may be specifically recognised by protective, anti-schistosome immune responses [55,56] and imply that SmVAL1, SmVAL4, SmVAL10 and SmVAL18 proteins are potential *S. mansoni *vaccine candidates.

Although we report the identification of twenty-eight SCP/TAPS family members in this study, the complete number of SmVAL genes will be unknown until the final genome build is released. Preliminary evidence that one of the seven incomplete SCP/TAPS genes (smp_120670) is transcribed (data not shown) does suggest more SmVAL (with complete SCP/TAPS domains) genes will be identified as the genome assembly progresses. The completion of the genome will also help in solving questions relating to SmVAL gene duplication events. During the sequencing of both cDNA and gDNA, we have identified two different SmVAL8 transcripts, two different SmVAL7 genomic sequences and evidence for at least four variations of the SmVAL3/SmVAL23 family (data not shown). The FISH localisation of the SmVAL2/SmVAL8/SmVAL12 BAC clone to more than two chromosomal regions (Fig. 6C) also seems to provide evidence for duplications events, although recent reports of non-specific BAC clone hybridization to the short arm of chromosome W have been reported [57]. Additionally, within the twenty-eight SmVALs, there are several that display such striking sequence conservation that a recent gene duplication event is the most likely explanation. For example, genes encoding SmVAL26 and SmVAL27 (both transcriptionally confirmed, Table 2) are 90% identical over encoded amino acid sequences (182AAs) and 93% identical over complete genomic sequences. This high level of identity over both exons and introns, suggests such genes have been through recent gene duplication events and supports the contention that the SmVAL family was expanded from two ancestral genes containing group-specific intron/exon structures (Fig. 4).

In the absence of biological functions for the SmVAL family, it is currently unknown why gene duplications have persisted. However, arguments can be made for all three of the main evolutionary conditions for copy retention – beneficial increased production, subfunctionalisation or neofunctionalisation [58]. The extra amount of an SmVAL gene product gained by two gene copies could be advantageous despite functional redundancy between the two proteins. This has been suggested for gene copies of *S. mansoni *cercarial elastases, which all appear to encode proteins of the same substrate specificity [59]. Subfunctionalisation describes a scenario where a protein encoded by the ancestral gene either has two biological functions or is expressed in two separate locations (physically or temporally). After the duplication event, each copy can then specialise in a unique function or in a distinct location. Neofunctionalisation describes a situation where one gene retains the original function while the other evolves a new function. Both subfunctionalisation and neofunctionalisation could explain at least one SmVAL gene duplication event, that of the ancestral SmVAL1/SmVAL2 gene. SmVAL1 and SmVAL2 are very similar genes (85% genomic identity) but have radically different expression profiles (Fig. 7) with SmVAL1 associated with vertebrate invasion and SmVAL2 associated with invertebrate invasion. One hypothesis is that the ancestral SmVAL1/SmVAL2 gene was expressed both in the cercaria and the miracidia, and carried out the same function during both invasive processes. After the duplication event SmVAL1 expression became restricted to cercaria and SmVAL2 became specialised for the miracidial function. Alternatively, the SmVAL1/SmVAL2 ancestral gene may have been expressed in only one invasive stage and gene duplication led to one gene gaining a new function (though it could possess a very similar biological activity) in the other invasive stage. Further work will be needed to dissect the exact mechanism responsible for SmVAL genomic expansion.

Previous results utilizing DNA microarrays demonstrated SmVAL6 and SmVAL7 to be male-associated in the adult worm [6]. The quantitative PCR assays reported here confirmed this finding with transcript levels 36-fold (SmVAL6) and 3-fold higher (SmVAL7) in adult males compared to adult females. Quantitative PCR also showed an adult female-biased expression for SmVAL13 mRNA (7-fold over adult male worms). Interestingly, several SCP/TAPS proteins in other species are also gender-associated. These include the mammalian CRISP-1 and CRISP-2 proteins, which are localised to the developing sperm and the *Xenopus *Allurin protein, which is associated with the egg [37,60]. Semi-quantitative reverse transcription PCR of the *Drosophila *SCP/TAPS family members also showed that the majority of the transcripts to be gender-associated with 18/26 showing male-associated expression and only one exhibiting female-associated expression [7]. Future experiments identifying SmVAL protein localisation within the adult worms will elucidate whether the connections between SCP/TAPS proteins with reproductive organs exist in the schistosome or the gender-associated transcription is due to other developmental differences.

A final observation of this study was the high level of developmentally-regulated alternative splicing found within the region encoding the long C-terminus of the group 2 member SmVAL6 (Fig. 8). The complex gene structure encoding the SmVAL6 C-terminus (Fig. 5) allows for alternative splicing of many small exons each in the same phase, avoiding frameshifts. Though the relevance of both the long C-terminus and the diversity observed within it is unknown, the developmental regulation of exon 20 and 26 suggests that the amino acid composition within the C-terminal region may be important for SmVAL6 function. Developmentally regulated alternative splicing occurs for a few other *S. mansoni *genes such as CA150 and SmHSF [61,62], although these examples result in the introduction of premature stops changing any resulting protein radically, unlike the small changes of exon 20 and 26 within SmVAL6. One potential biological difference between cercarial and adult worm SmVAL6 transcripts is in phosphorylation. Exon 20 and 26 (statistically differentially expressed in the adult, Fig. 7) encode highly similar amino acid pentamers (KDDQY and KDEQY respectively) that are predicted to be phosphorylated at the tyrosine residue (data not shown). If these are functional phosphorylation sites, many SmVAL6-associated biological processes (protein-protein interactions, protein location within the cell, increased protein degradation and signalling) could be differentially affected in the cercarial versus the adult life stage. Interestingly, group 2 GAPR-1 can be differentially phosphorylated and this phenomenon has been associated with different cellular locations [63]. Further work to ascertain the relevance of SmVAL6 developmentally-regulated splicing, as it applies to function, is ongoing.

## Conclusion

In this study, we identified twenty-eight *S. mansoni *genes encoding proteins containing SCP/TAPS domains, named SmVAL1-28. Examination of protein features and gene structures has revealed two distinct groups within the SmVAL gene family and phylogenetic analysis demonstrated both group 1 and group 2 type SCP/TAPS proteins to be present across the metazoan sub-kingdom. We also obtained developmental expression profiles of SmVAL1-13, which suggested that several of the proteins are involved in host invasion. Furthermore, we have discovered developmentally regulated alternative splicing within the 3' region of SmVAL6. Further work is aimed at functionally characterising this interesting schistosome protein family in terms of host/parasite and parasite/parasite interactions.

## Methods

### Parasite Materials

A Puerto Rican isolate of *Schistosoma mansoni *is maintained by passage through *Biomphalaria glabrata *snails and Tuck Ordinary (T.O.) mice (Harlan). The fifteen lifestages (mixed sex unless stated) providing RNA for Real-time quantitative developmental expression analysis were: Egg; Miracidia; Mother sporocyst; Cercariae; 3-hour schistosomule; 18-hour schistosomule; 3-day schistosomule; 6-day schistosomule; 3-week worm; 4-week worm; 5-week worm; 6-week worm; 7-week worm; 7-week male, and 7-week female.

Eggs were recovered from mouse livers infected 7 weeks prior with cercariae as previously described [64]. To isolate miracidia, the eggs were then hatched in non-chlorinated water and miracidia were separated phototrophically. Mother sporocyst RNA was provided by Dr. Tim Yoshino, of the University of Wisconsin [65]. Cercariae were shed from snails phototrophically, with schistosomula prepared by mechanical transformation of cercariae as previously described [66]. Schistosomula were cultured at 37°C in Dulbecco's Modified Eagle's medium (DMEM) (Sigma, UK) supplemented with 10% FCS and 100 ng/ml penicillin-streptomycin for the time periods indicated in an atmosphere of 5% CO_2 _[66]. Adult worm lifestages (3 wk-7 wk) were obtained by perfusion at the stated week from percutaneously infected T.O. mice after challenge with 250 cercariae. After perfusion, worms were counted and, in the case of 7 wk adults, separated on the basis of gender.

### Total RNA isolation and cDNA synthesis

Total RNA was isolated using a modified TRIZOL (Invitrogen)/RNeasy (Qiagen) procedure [67] and subsequently treated with DNase I (Ambion Inc.) to remove contaminating genomic DNA. RNA quality from each sample was assessed by formaldehyde denaturing gel electrophoresis and quantity determined by spectrophotometry at 260 nm.

Total RNA from the fifteen lifecycle stages were used as templates to synthesise cDNA by reverse transcription as described previously [68]. The efficiency and quality of the cDNA obtained was tested using an alpha-tubulin (SmAT1) specific primer-pair [69] to amplify SmAT1 cDNA by polymerase chain reaction (PCR).

### cDNA and gene sequence determination

Using *S. mansoni *Gene DB [24], the complete open reading frames (ORFs) of 28 SmVAL genes were predicted (SmVAL1-28). The sequences of SmVAL1-13 ORFs were confirmed by PCR amplification from parasite cDNA using proof-reading enzymes (HiFi Platinium Taq (Invitrogen) or Phusion (GRI)), cloned into pCR4-TOPO vector (Invitrogen) and sequenced. In addition, Rapid Amplification of cDNA ends (RACE) PCR was performed for selected SmVALs using RNA from parasite lifestages processed into cDNA according to the manufacturer's protocol (GeneRacer kit, Invitrogen). The ORF sequences for SmVAL1-13 have been submitted to Genbank, accession numbers are shown in Table 1. Additional *S. mansoni *Gene DB interrogation using the Pfam [26] HMM model for SCP/TAPS domain (PF00188) resulted in the discovery of a further 15 genes (SmVAL14-28) predicted to encode full SCP/TAPS domains and 7 predicted to encode incomplete SCP/TAPS domains (Table 2). PCR amplification from parasite cDNA and sequencing of the resulting fragments confirmed transcription for 11 of the 15 gene products containing full SCP/TAPS domains.

The genomic sequences of all SmVAL genes (start to stop codons, including nucleotide discrepancies 'N', where each 'N' was treated as an individual nucleotide) were derived from analysis of *S. mansoni *Gene DB version 4 with regions of genomic sequences for SmVAL 1-13 confirmed by PCR amplification. The genomic DNA used for PCR amplification of SmVAL1-13 was purified from *S. mansoni *adult worm and cercarial material according to the protocol provided with the DNeasy Tissue kit (Qiagen). Sequencing of the SmVAL cDNAs or gene fragments was performed at the Department of Genetics, University of Cambridge in both orientations using Big Dye v3.1 fluorescent chemistry and an Applied Biosystems 3100 Genetic Analyser.

### Bioinformatic Analysis

Domain prediction was performed by the SMART sequence analysis software [27] and was used, with manual alignment comparison, to define the limits of the SCP/TAPS domain for each sequence.

The prediction of signal peptides was performed using the software SignalP 3.0 [28] and presence/absence of signal peptides was defined by the default Neural Network *D-score *threshold of 0.43.

### Alignment and Phylogenetic trees

Alignment of the twenty-nine SCP/TAPS domains from the 28 SmVALs was created using MUSCLE software [70]. For comparison of sequence conservation within *S. mansoni *to a wide range of SCP/TAPS domain containing proteins, a consensus sequence was generated using the family alignment for the SCP/TAPS domain (SM00198) found in the SMART database [27].

For the phylogenetic analysis of the SmVAL family and other metazoan SCP/TAPS family members, SCP/TAPS domain regions were aligned using MUSCLE software and interrogated to determine regions of conservation using GBLOCKs 0.91b software [30]. For the SmVAL phylogenetic analysis six such regions covering a total of 84 amino acids were chosen, whereas the metazoan analysis used four regions containing a total of 54 amino acids. The phylogenetic analyses were performed as described previously [69] using MrBayes (version 3.1.2 [71]) software and the WAG protein substitution model [72]. The consensus phylograms were visualised using the TreeView 1.6.6 program [73]. Utilizing the same alignment and conserved regions, phylogenetic trees were also constructed using the Neighbor-Joining method and the Poisson correction model, accompanied by bootstrap analysis (1000 replications). The trees were produced by MEGA v4.0 [74].

### Homology modelling

The Na-ASP 2 (pdb: 1u53[13]) and p14a (pdb: 1cfe[8]) structures were used as templates to build models for group 1 SmVAL proteins. The GAPR-1 structure (pdb: 1smb,[10]) was used as the template for the creation of group 2 SmVAL protein models. An alignment of the protein sequences was initially generated by MUSCLE software and refined manually. Models were created using MODELLER software [31] and the validity of the models chosen were tested using WHATCHECK and PROSAII [75,76]. Models were visualized using MacPyMOL (DeLano Scientific LLC).

### FISH

*S. mansoni *Gene DB version 1 was used to identify BAC clones (obtained from the Sm1 library [77]) containing SmVAL gene clusters. The presence of SmVAL2, SmVAL8 and SmVAL12 on Sm1-41J19 was confirmed by PCR amplification, agarose gel electrophoresis and automated DNA sequencing. Fluorescence *in-situ *hybridisation (FISH) was used to identify the chromosomal localisation of BAC clone Sm1-41J19, using a biotinylated probe made from the BAC clone. The probe was hybridised to *S. mansoni *chromosomes using established procedures [78].

### Real-time RT-PCR analysis

The transcript abundance of each sequence-verified SmVAL (SmVAL1-13) mRNA was quantified relative to SmAT1 (alpha tubulin) in fifteen stages of the *S. mansoni *lifecycle using real-time RT-PCR analysis. The cDNA from 15 different life stages was assayed by real-time PCR in duplicate reactions using gene-specific primers. To confirm the absence of non-specific amplification in PCR reactions, control reactions lacking template were run in duplicate for each primer set. Reactions were performed on a MiniOpticon real-time PCR thermal cycler system (Bio-Rad) using iQ SYBR Green Master Mix (Bio-Rad) according to the manufacturer's instructions. The amplification efficiency (E) of each primer set was determined during assay development by plotting the cycle thresholds (C_t_) from serial dilutions of a suitable cDNA sample and inputting the resulting slope in the equation, E = 10^(-1/slope)^. For each real-time PCR reaction the following equation was used to calculate a normalised SmVAL expression ratio:

ratio = (E_SmAt1_)^CtSmAT1^/(E_SmVALx_)^CtSmVALx^

where E_SmAt1 _is the amplification efficiency of the reference gene (alpha tubulin), E_SmVALx _is the amplification efficiency of the target gene (SmVAL), CtSmVALx is the cycle threshold of the target gene and CtSmAT1 is the cycle threshold of the reference gene from the same cDNA sample [79].

### SmVAL6 splicing study

A primer pair (VAL6 var F/VAL6 var R) designed to amplify the region containing all identified variability discovered in previous RACE and ORF sequencing of SmVAL6 transcripts was used to examine SmVAL6 sequence variation (see Fig. 5 for primer locations). PCR reactions using this primer pair with either cercarial or 7 wk mixed sex worm cDNA were carried out using Platinum High Fidelity Taq polymerase (Invitrogen). The PCR products were ligated into the pCR2.1 vector (Invitrogen). Thirty-two 7-week adult clones and thirty-five cercarial clones were picked at random and fully sequenced. The sequences were then aligned to ascertain the exon usage of each clone. Student's χ^2 ^test was used to determine any significant associations between exon usage and developmental life stage.

## Authors' contributions

IWC, AJM and MAW cloned the SmVAL transcripts and gene fragments, HH performed the FISH experiments, RS helped create the homology models and RMRC identified homologous SCP/TAPS metazoan sequences. IWC analysed the results. KFH designed the study. IWC and KFH wrote the paper. All authors read and approved the final manuscript.

## Supplementary Material

Additional file 1 Neighbor-Joining phylogenetic tree derived from SmVAL protein sequences. Phylogenetic relations were examined using Neighbor-Joining method under the Poisson substitution model. Support values for the consensus tree were obtained by bootstrapping 1000 replicates and are shown. Branch length represents the number of amino acid changes calculated by the Poisson model and is indicated by the scale bar. Click here for file
