# Supplementary material for: Developmentally regulated expression, alternative splicing and distinct sub-groupings in members of the Schistosoma mansoni venom allergen-like (SmVAL) gene family
Source: BMC Genomics. 2008 Feb 23;9:89. doi: 10.1186/1471-2164-9-89 (PMC2270263; doi:10.1186/1471-2164-9-89)
Supplement: Supplementary file 1 [file 1471-2164-9-89-S1.DOC]

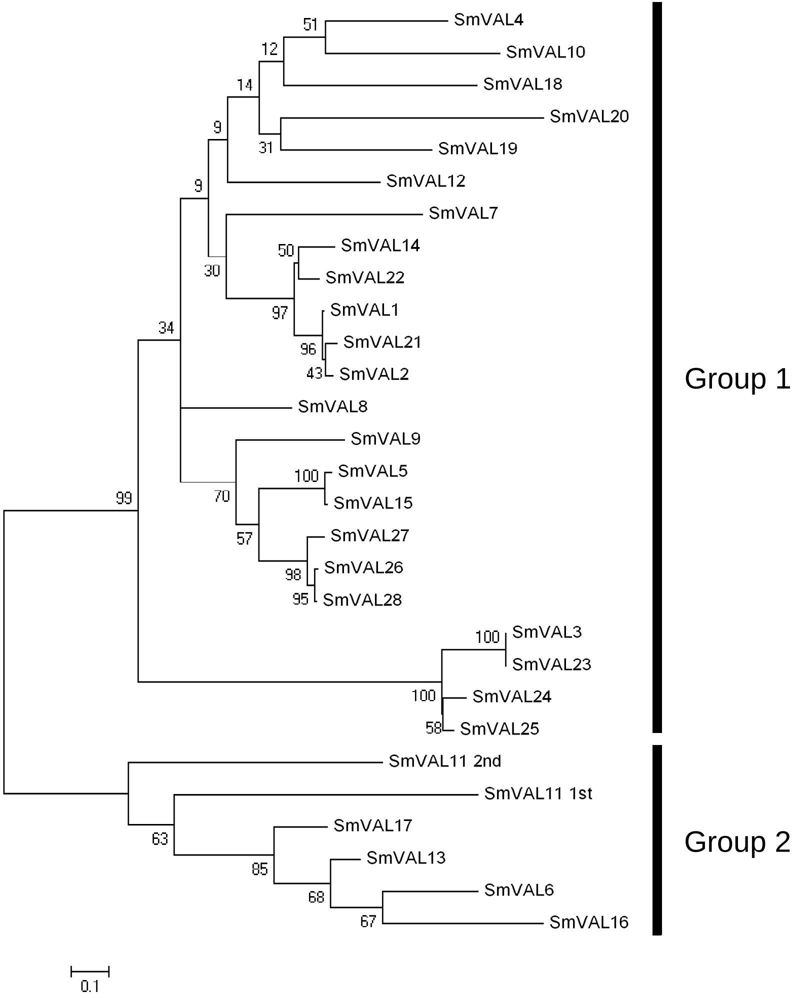

SmVAL4
SmVAL10
SmVAL18
SmVAL20
SmVAL19
SmVAL12
SmVAL7
SmVAL14
SmVAL22
SmVAL1
SmVAL21
SmVAL2
SmVAL8
SmVAL9
SmVAL5
SmVAL15
SmVAL27
SmVAL26
SmVAL28
SmVAL3
SmVAL23
SmVAL24
SmVAL25
SmVAL11 2nd
SmVAL11 1st
SmVAL17
SmVAL13
SmVAL6
SmVAL16
51
12
14
9
31
9
50
30
97
34
96
43
100
70
99
57
98
95
100
100
58
63
85
68
67
0.1
Group 1
Group 2
